# Exploring the value and role of integrated supportive science courses in the reformed medical curriculum iMED: a mixed methods study

**DOI:** 10.1186/s12909-016-0646-9

**Published:** 2016-04-29

**Authors:** Sophie Eisenbarth, Thomas Tilling, Eva Lueerss, Jelka Meyer, Susanne Sehner, Andreas H. Guse, Jennifer Guse (nee Kurré)

**Affiliations:** Department of Biochemistry and Molecular Cell Biology, University Medical Center, Hamburg, Germany; Dean’s Office of Education and Students’ Affairs, University Medical Center, Hamburg, Germany; Department of Medical Biometry and Epidemiology, University Medical Center, Hamburg, Germany; Department of Medical Psychology, University Medical Center, Hamburg, Germany

**Keywords:** Curriculum reform, Science education, Integration, Medical education

## Abstract

**Background:**

Heterogeneous basic science knowledge of medical students is an important challenge for medical education. In this study, the authors aimed at exploring the value and role of integrated supportive science (ISS) courses as a novel approach to address this challenge and to promote learning basic science concepts in medical education. ISS courses were embedded in a reformed medical curriculum.

**Methods:**

The authors used a mixed methods approach including four focus groups involving ISS course lecturers and students (two each), and five surveys of one student cohort covering the results of regular student evaluations including the ISS courses across one study year. They conducted their study at the University Medical Center Hamburg-Eppendorf between December 2013 and July 2014.

**Results:**

Fourteen first-year medical students and thirteen ISS course lecturers participated in the focus groups. The authors identified several themes focused on the temporal integration of ISS courses into the medical curriculum, the integration of ISS course contents into core curriculum contents, the value and role of ISS courses, and the courses’ setting and atmosphere. The integrated course concept was positively accepted by both groups, with participants suggesting that it promotes retention of basic science knowledge. Values and roles identified by focus group participants included promotion of basic understanding of science concepts, integration of foundational and applied learning, and maximization of students’ engagement and motivation. Building close links between ISS course contents and the core curriculum appeared to be crucial. Survey results confirmed qualitative findings regarding students’ satisfaction, with some courses still requiring optimization.

**Conclusions:**

Integration of supportive basic science courses, traditionally rather part of premedical education, into the medical curriculum appears to be a feasible strategy to improve medical students’ understanding of basic science concepts and to increase their motivation and engagement.

**Electronic supplementary material:**

The online version of this article (doi:10.1186/s12909-016-0646-9) contains supplementary material, which is available to authorized users.

## Background

The heterogeneity regarding ethnicity, culture and social background in medical school students becomes more and more relevant for the development of curricula [1]. In parallel, a notable variance in science knowledge is observed [2]. Prior research has focused on the problems that arise from this situation, e.g., dropout of students [3]. Coping with this complex situation is a challenge for universities worldwide. There are different ways medical schools support their students. Pre-matriculation programs are widespread in the US [4–6] as a possibility for students to be prepared in basic sciences for medical school before starting. In contrast, remediation interventions [7] are only applied after students performed poorly in exams. Both offers have in common that they concentrate on low-performing students, being “at risk”. Another approach to support students is to offer introductory courses in sciences for all first-year students immediately before classes start, a common practice in medical schools in Germany. This ‘en bloc’ approach, however, does not take into account findings showing that basic science contents can be better recalled and transferred when embedded in clinical topics [8].

Thus, medical schools worldwide try to overcome the curricular separation between basic medical and clinical sciences [9–17]. This has resulted from applying Flexner’s model [18] to medical curricula since the early 20th century. Although several medical schools have published their experiences with integrated curricular since then [19–22], there is still an ongoing debate about the integration of basic sciences [14, 23], and a demand for reforming medical and premedical education [24, 25]. More specifically, there have been no reports on new curricula that use that kind of integrated approach for supportive science courses (ISS courses) so far. Recently, the University Medical Center Hamburg-Eppendorf has been implementing such an innovative concept in conjunction with a reformed medical curriculum termed iMED.

### Integrated supportive science courses

**Fig. 1:**
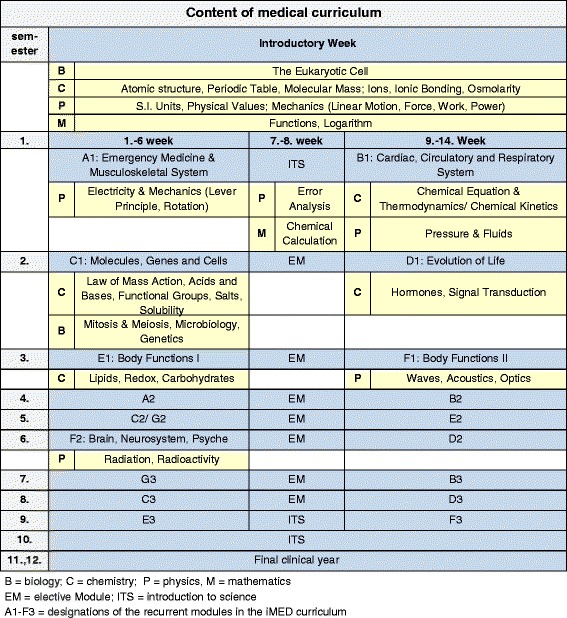
Integrated medical curriculum iMED including ISS courses. Scheme depicting the modular structure of the integrated medical curriculum iMED at the University Medical Center Hamburg-Eppendorf, including the temporal locations and contents of supportive science (ISS) courses

The ISS courses were launched in 2012 contemporaneously with the reformed curriculum iMED at the University Medical Center Hamburg-Eppendorf. Each year 380 students are enrolled in the medical school. The aim of the ISS courses is to bridge the gap between students’ previous science knowledge and the requirements of medical school and to specifically improve their understanding of science (biology, chemistry and physics) and mathematics. The ISS courses were taught on a higher school level, they were voluntary and offered to all students. Each course has a maximum group size of 20 students and duration of 90 min. The matching of the ISS courses with the iMED curriculum with respect to content and time is shown in Fig. 1.

The curriculum structure itself provides horizontal and vertical integration [14]. Modules are usually structured by common diseases; these diseases are used to teach matching basic and clinical topics by different disciplines. According to Harden’s ladder of integration [26], the iMED curriculum corresponds to a multi-disciplinary approach as well as to the principles of reform curricula [27]. One week before the start of the first semester, basic knowledge of all four ISS course classes is taught in a block (*introductory week*). The content of these courses prepares for the contents of the core curriculum in the first module (first half of first semester). After the introductory week, we implemented a time-wise and content-based integration of all ISS courses into the first modules of the medical curriculum. For example, in the module *B1*, physical basic knowledge (pressure, fluids, flow) is the subject of two ISS courses while Cardiac, Circulatory and Respiratory System is the topic in physiology and other core curriculum subjects (see Fig. 1).

### Aims of the study

Unlike other aspects of medical education, little research has focused on introductory science course programs in medical education so far. In particular, the possible integration of such courses into the curriculum has not been explored. Thus, we set out to investigate the value and role of our innovative ISS courses: What is the role and value of the ISS courses? How are the ISS courses accepted? What can we say about participation of the ISS courses? As a new curriculum is always subject to continuous optimization, a major aim for us was the success of integration in terms of students’ participation and satisfaction: What has worked out well or what should be improved in the future? To get an impression from different points of view, we examined the perception of both lecturers and students.

Additionally we wished to explore whether students report different ratings of the course items over time, analysed separately for the four classes (chemistry, biology, physics, mathematics).

## Methods

This was a single-center study with a mixed-methods design including qualitative and quantitative data [28]. The study was conducted at the University Medical Center Hamburg-Eppendorf between December 2013 and July 2014. To obtain qualitative data the authors conducted separate focus groups with students of cohort 2013 and lecturers of the ISS courses. Quantitative data were collected from all students of cohort 2013 responding to end-of-module surveys during the first study year.

### Participants and setting

#### Focus groups

All lecturers and all first-year students of the cohort 2013 who attended ISS courses at least once were invited via email to participate in the focus groups. In addition, the invitation for first-year students was also provided during a lecture.

For both students and lecturers, two focus groups each took place in May 2014 in quiet rooms of the University Medical Center Hamburg-Eppendorf. Prior to focus group discussions, participants were asked to answer a short socio-demographic questionnaire. Additionally, students were asked to rate their satisfaction with the ISS courses on a 1-6 Likert scale ranging from “strongly disagree” (1) to “strongly agree” (6). For lecturers we included one item to specify their teaching experience and categorized it as follows: 0–2 years, 3–5 years, 6–9 years, 10 and more years teaching experience. Besides, informed consent was received before the focus group sessions started. Each of the focus groups had a length of 45–120 min and was moderated by a psychologist. A second moderator commented the consensus within the focus group in a pre-assembled matrix [29].

The authors developed drafts, using the results of Finnerty et al. [18], and conducted a pretest with four students in May 2014. All students taking part in the pretest had participated in the ISS courses one year before. Questions were refined after the pretest and included the following final items: *“What is the value and the role of the ISS courses in medical education?”, “How did you experience the atmosphere in the ISS courses?”, “How do you assess the knowledge retention of the contents taught in the ISS courses? How could it be influenced?”, “When and how should the ISS courses be incorporated into the medical education curriculum?”, “What is the value of teaching in small groups?”.*

Each focus group session was audiotaped and the recordings were transcribed verbatim.

#### Survey

Longitudinal data on the students of the cohort 2013 who provided responses to the online survey at the end of each module of the medical curriculum were evaluated. During their first academic year students completed five end-of-module surveys. The surveys cover all ISS courses of the first study year. The first question of the survey referred to participation in the ISS courses to ensure that only participating students rated the courses. Participation in ISS courses is defined as answering “yes” to this question. Non-participating students were excluded from further evaluation. Three authors (SE, TT and JM) developed study specific questions to explore students’ satisfaction with the ISS courses and their helpfulness. The questionnaire was reviewed and approved by all authors to verify that questions were understandable and clear and is available online as Additional file 1. The items were rated on a 1–6 Likert scale ranging from “strongly disagree” (1) to “strongly agree” (6).

### Data analysis

#### Focus groups

Qualitative research was performed in accordance with the consolidated criteria for reporting qualitative research (COREQ) of Tong et al. [30]. We used conventional content analysis with inductive categorizing as recommended by Hsieh and Shannon [31]. Two independent investigators (EL, SE) started with free reading of the transcripts. Emerging themes were coded and sorted to categories, which were reviewed multiple times by the authors until consensus was reached. The software used was *MAXQDA 10*.

#### Survey

Descriptive statistics were generated to provide an overview. Age was categorized as two categories (≤25 years or > 25 years). A non-responder-analysis was performed, comparing participants and non-participants of the ISS-courses for gender and age, depending on class and module, using multiple regression models. In a stepwise backward elimination, variables were selected by using Wald Chi-Square Test. The first model included all four variables and the interactions between module and participants characteristics (age, gender) as well as class and participants characteristics as predictors. In a stepwise backward elimination, variables were selected by using Wald Chi-Square Test. For the analysis of the both items we used a linear mixed model with questionnaire as random effect to adjust for the cluster structure in the data. Interesting fixed predictors were items, modules and classes and especially their three-way-interaction. The model was further adjusted for age and gender. Adjusted means and 95 % confidence intervals were reported. The level of significance was set at alpha = 0.05. All statistical analyses were conducted using IBM SPSS Version 20.0.

### Ethical approval

The study was carried out in accordance with the Declaration of Helsinki. The anonymity of participants is guaranteed and participation was voluntary. The study was fully explained and all participants gave written informed consent. At the time we were seeking ethical approval for our study there was no institutional ethics committee. The Dean of the University Medical Center Hamburg-Eppendorf was authorized qua function to substitute the institutional ethics committee and approved the study.

## Results

### Focus groups

Of 14 first-year medical students, who attended the student focus groups, 50 % (*n* = 7) were female. The mean age was 21.6 years (SD = 2.3). They reported a mean satisfaction with the ISS courses of M = 4.6 (SD = 0.5). 13 were German and one was Italian. Among 13 lecturers participating in the focus groups 15.4 % (*n* = 2) were female. The mean age was 44 years (*SD* = 10.6). 12 were German and one was Czech. Teaching experience was 0–2 years (5 lecturers, 38.5 %), 3–5 years (1 lecturer, 7.7 %), or more than 10 years (5 lecturers, 38.5 %). For two lecturers, data regarding age and teaching experience are missing.

We determined several themes describing the value and role of the ISS courses, setting and atmosphere of these courses, integration of the courses into the medical curriculum in respect to time and content, and ideas for improvement of ISS courses. We found high accordance between the statements of lecturers as well as of students.

### Value and role of the ISS courses

Five subthemes emerged from the focus groups representing the value and the role of the supportive science courses for medical students: maximization of students’ engagement and motivation, increase of knowledge retention, creation of a basic understanding, integration of foundational and applied learning, and alignment of students’ proficiency level. Students and lecturers said that a deepened understanding results in a maximization of students’ engagement and motivation. One student stated:

*“My personal motivation was: I don’t want to learn the relevant topics by heart only, but I want to understand them. To this end, the ISS courses were extremely helpful”.*

Additional students reported that refreshment and repetition of science contents from school maintained their motivation. One student emphasized that in-depth study increased students’ motivation:

*“We have deepened the interesting topics in the small groups. We left the course really motivated or even enthusiastic about the topic”.*

Several students mentioned that motivated lecturers who connect medical knowledge with science contents had maximized their personal motivation.

Many lecturers and students said that knowledge retention of the ISS course contents is increased by repetition of contents. In addition to that, some students mentioned applied exercises performed in the ISS courses as a way to increase the retention of the science knowledge.

Participants of all focus groups considered the creation of a basic understanding as a value of the ISS courses, which is achieved by improving basic knowledge and recognising connections. One student pointed out:

*“In ISS courses it’s about the whole, so that I understood the context in the lecture course. I thus got a more profound understanding, which has an impact on my performance”.*

Likewise, one lecturer stated:

*“For me, the value of the ISS courses is that the students acquire a fundamental understanding of the subject. And that they do not rely on learning facts by heart, but that they have the competence to classify the facts into a system. And that they build up a system during their study, into which the clinical contents can be incorporated”.*

Students and lecturers reported that the basic understanding can also be attained by testing the knowledge by answering questions in the ISS courses. Some students underlined that participation in several ISS courses was helpful to pass examinations.

Another value of the ISS courses reported by both lecturers and students is the integration of foundational and applied learning. They stated that scientific knowledge taught in these courses is required not only for the medical school, but also for later work as a physician. One student noted:

*“I think that you should expect more of your courses than only to pass. As a physician you need background and understanding. And I think that this is well imparted in the ISS courses, because they offer a different form of learning”.*

Many participants noted the alignment of students’ proficiency level as an important role of the ISS courses. One student said:

*“For instance, I did not have a lot of physics at school. My expectancy was that the lecturers try to meet the students at each ones ground and to bring them to the same level.”*

### Setting and atmosphere

All focus group participants, lecturers as well as students described the atmosphere in the ISS courses as “friendly” and “relaxed”. Two main topics related to the setting and atmosphere emerged from the focus groups: Learning in small groups and the voluntary participation in the ISS courses. Lecturers and students accentuated the effects of learning in small groups, which reduces inhibition, is learner-centered and enables the formation of study groups.

In addition, the voluntary participation in the ISS courses was intensely discussed in all focus groups. Both lecturers and students reported high intrinsic motivation of participating students. Some students pointed out that the voluntariness promotes their personal responsibility and self-organization (see Table 1 for representative quotes).

**Table 1:** Subthemes and representative quotes for the theme “Setting and atmosphere of ISS courses” from focus groups with students (*n* = 14) and lecturers (*n* = 13) at University Medical Center Hamburg-Eppendorf

Focus group	With lecturers (*n* = 13)	With students (*n* = 14)
Subthemes	Representative quotes	Representative quotes
Teaching in small groups • Reduces inhibition • Is learner-centered • Enables formation of study groups	Reduces inhibition: *“There is a big advantage for weak students. They know: “In the ISS courses we are amongst each other, and now I dare to ask a seemingly stupid question”.* Learner-centered: *“It is nice that the ISS courses are not affected by the pressure of an exam. Thus, the lecturer is able to react more flexibly to the students” questions, wishes and needs than in the core curriculum”.* Enables formation of study groups: *“Learning in small groups is good for team building and for the ability to discuss with each other”.*	Reduces inhibition: *“For me it’s difficult to ask questions in front of four hundred people during a lecture when I have trouble following. In contrast, in the ISS courses, I can ask more specific questions if I want to know more”.* Learner-centered: “*I liked that one could also shape the conversation and that it was not necessarily focused on the scheduled topics, but that we rather checked “Where are we now? Is there still need to repeat, or could we dig in a little deeper?”* Enables formation of study groups: *“The ISS courses often are like study groups. Explaining [things to] each other, helping each other. I’m a fan of study groups”.* *“In this case, the study group is like a speaking textbook”.*
Voluntary participation: • Participation of interested students • Encourages the students to express their demand • Promotes personal responsibility and self-organization • Disadvantage: Not all students of the target group participate.	Participation of interested students *“Students who attend the ISS courses are interested and dedicated. This has a positive effect on the atmosphere and on the whole [learning] progress”.* Encourages the students to express their demand: *“Students who participate in the ISS course more pronouncedly articulate their needs”.* Disadvantage: Not all students of the target group participate: *“A disadvantage is that some students who could benefit from the courses miss the chance because it’s voluntary”.*	Participation of interested students *“Because the ISS courses were voluntary, only interested people attended. Those who were there were all motivated and wanted to learn something”.* Promotes personal responsibility: *“Having courses to choose from independently is an advantage because you get the feeling that you are granted individual responsibility”.* Promotes self-organization: “*In our core curriculum, we don’t have many choices, so I think it’s very important that students can decide by themselves how to organize their learning during the course of studying”.*

### Integration into the medical curriculum

All participants reported that matching of ISS courses and core curriculum was overall successful, with slight differences between content topics. One student told his experience in module B1:

*“In module B1 there was a great connection between the physiology lectures, the physics lectures picking up from there and the ISS courses that deepened the understanding”.*

One aspect of integration discussed in the focus groups was the timing of the ISS courses. In general, both students and lecturers reported a close connection between curricular and extracurricular contents and appreciated the integrated concept of the ISS courses in comparison with the traditional en bloc teaching. One student said:

*“I think the best is that the ISS courses match the modules’ contents and that there is not just a one week preparation class at the beginning of the medical school”.*

Moreover, one lecturer argued that knowledge retention is low without the need for practical application, referring to the preparation class before the first semester, which was offered in the traditional curriculum.

There was a broad consensus among students regarding the advantage of a close temporal connection between ISS courses and core curriculum.

Many lecturers and students said that the knowledge retention of the ISS course contents is increased by a tight temporal link with the curricular medical training. In this context, one lecturer stated:

*“Knowledge retention is directly dependent on how close the connection is to the curricular medical training. The better the connection, the more knowledge remains”.*

The second important aspect of integration mentioned by focus group participants is the connection between ISS course contents and core curriculum contents. Here, students named several advantages of the ISS course concept, referring to the importance of basic knowledge, knowledge retention and dealing with relevant topics.

First, students reported that they found the ISS courses to be a useful introduction at the beginning of a module, being prepared for what they have to learn in the near future. With regard to this aspect, one student added:

*“The lecturers aimed to show the importance of the [scientific] basics for each module”.*

In this context, one lecturer also pointed out the need for teachers of the ISS courses to refer to contents of the core curriculum as often as possible:

*“Often it is possible to refer to current things in the core curriculum. If not, there is no additional benefit for the students”.*

Second, students mentioned that, in comparison to the traditional preparation class, the new way of learning science basics in the ISS courses makes it easier to retrieve knowledge. One student explained:

*“Things to learn were split into small portions. That’s why it is easier to remember. There is not so much to learn at the same time”.*

Third, in addition to the above mentioned knowledge retention, students noted the possibility to discuss relevant topics intensely in study groups during the sessions and with the lecturer. One student added:

*“In contrast to a preparatory week, one focuses on small pieces of knowledge over a larger period of time. That makes it more efficient. There is more time to look into it”.*

### Ideas for Improvement

The participants of the focus groups, lecturers as well as students, outlined the advantage of additional exercises for optimizing the ISS courses, including more exercises with solutions during the ISS courses and typical examination questions at the end of each course.

Students proposed that lecturers should refer more often to core curriculum contents in the ISS courses, but also vice versa. Students suggested an orientation training for new lecturers to clarify the interface of ISS courses and core curriculum.

Additional students recommended including engaged students in the organization of the ISS courses, e.g., repetition session with experienced students as moderators or feedback given by students.

### Survey

Of 368 students (cohort 2013) who were enrolled at the end of the first academic year by the time of the last survey, 348 (94.6 %) students responded to the questionnaire. Among respondents, 58 % (*n* = 202) were female and 81.9 % (*n* = 300) were 25 years old or younger at baseline.

**Fig. 2:**
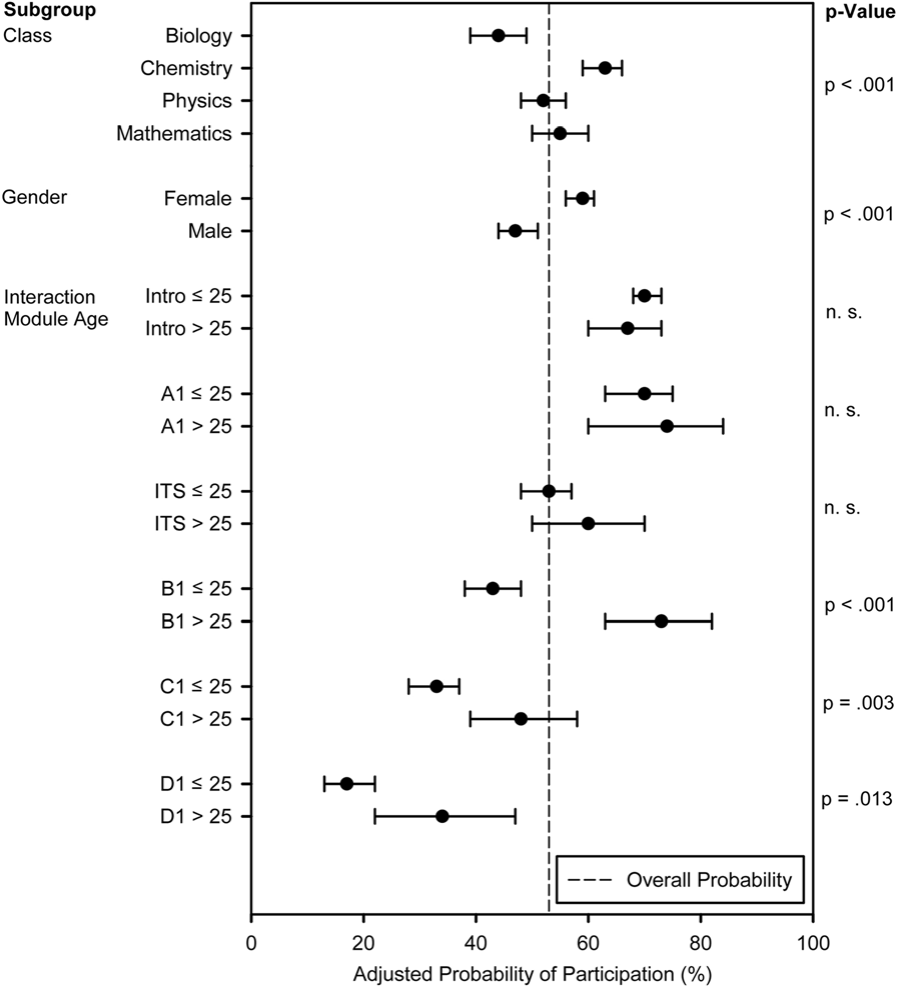
Student participation in ISS courses (cohort 2013). Probability of student participation in ISS courses during the first academic year at the University Medical Center Hamburg-Eppendorf (cohort 2013): a non-responder analysis was performed taking into account the parameters class, gender, age and module. Values are given as adjusted probabilities and 95 % confidence intervals. Intro = introductory week; ITS = introduction to science; A1, B1, C1, D1 = modules of the iMED curriculum

The non-responder-analysis included the interaction between module and age and the respective main effects as well as gender and class. It showed a significant influence of class (*p* < .001) and gender (*p* < .001) and a significant interaction between age and module (*p* < .001). Probabilities are shown in Fig. 2.

The overall participants rate is 53 % (95 %-CI [51 %; 56 %]. Pairwise comparisons showed significant differences between all classes (*p* < .05), except for the difference between physics and mathematics. The probability to attend the ISS courses was higher in chemistry compared to other classes. Female students participated significantly more often than male students did (*p* < .001). The participation of older students was significantly higher than of younger students in B1 (*p* < .001), C1 (*p* = .003) and D1 (*p* = .013). Participation decreased over time.

**Fig. 3:**
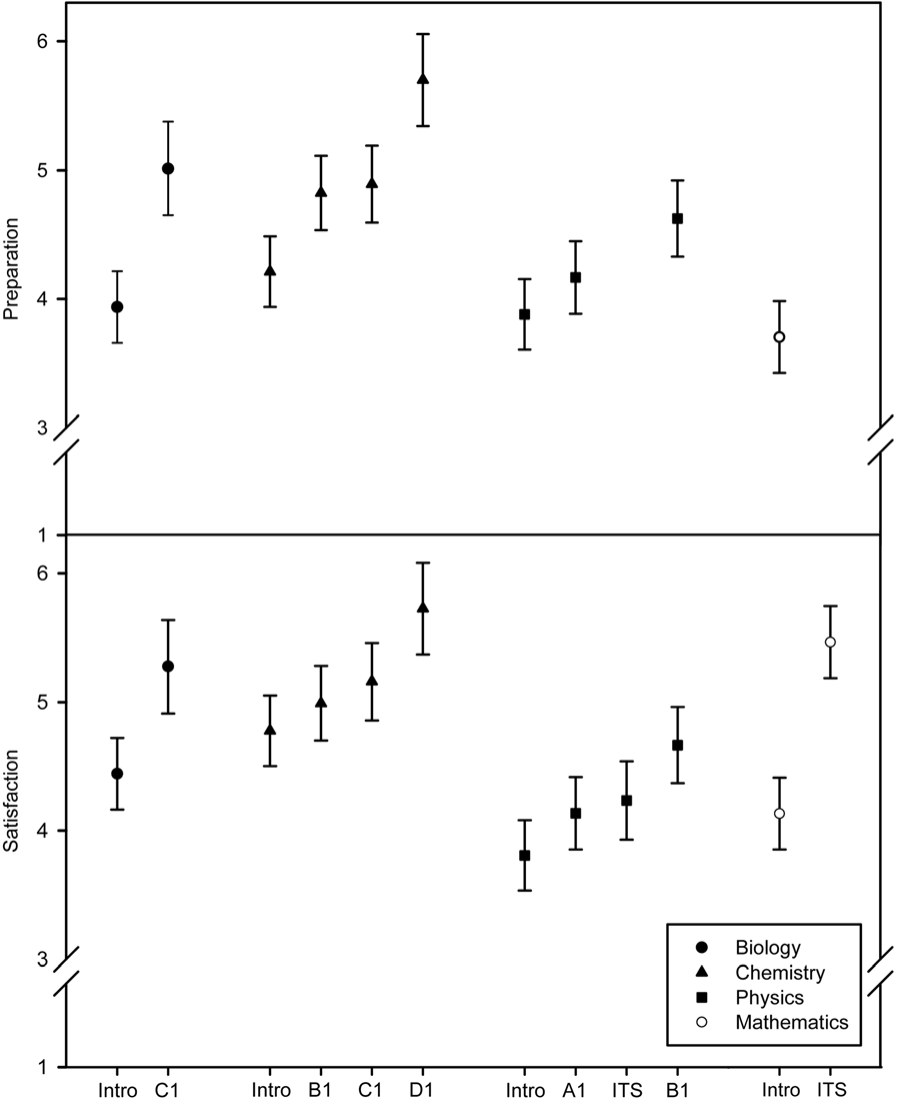
Students’ rating of ISS courses (cohort 2013). Comparison of students’ ratings of ISS courses during the first academic year (cohort 2013). The items “The ISS courses were helpful for preparing teaching units” (preparation) and “All in all, I am satisfied with the ISS courses” (satisfaction) were rated on a 1-6 scale ranging from “strongly disagree” (1) to “strongly agree” (6). Adjusted means and 95 % confidence intervals of the items are given for individual classes and modules. Intro = introductory week; ITS = introduction to science; A1, B1, C1, D1 = modules of the iMED curriculum

The survey comprised two items judged by first-year medical students during their regular evaluations at the end of modules: first, *“The ISS courses were helpful for preparing teaching units”* (*Preparation*) and second, *“All in all, I am satisfied with the ISS courses”* (*Satisfaction*)*.* All main effects and interaction effects were significant (*p* < .05). Pairwise comparisons for each class showed that both items were significantly better rated after the last ISS course in the study year compared to the first one in ITS (*p* < .001) (see Fig. 3 and Additional file 2).

## Discussion

More and more medical curricula aim to integrate basic biomedical classes, e.g., biochemistry and physiology, and clinical classes [14, 18, 20, 23, 24]. However, underlying basic sciences like physics or chemistry are less frequently integrated. The new supportive science (ISS) course concept at the Medical School of the University of Hamburg aims at such an integration of these basic sciences, addressing needs of an increasingly heterogeneous student population [1, 2]. Our study examines these ISS courses, taking a mixed methods approach including focus group discussions as well as quantitative data of end-of-module student evaluations. To our best knowledge, such ISS courses in medical education have not been explored so far.

Generally, the integration of ISS course contents into the curriculum, as well as the close temporal vicinity between ISS courses and corresponding core curriculum courses was positively received both in the lecturers’ and in the students’ focus groups. The focus group participants explicitly stated that the temporal integration of the ISS courses enhances the retention of science knowledge. In line with this statement, van der Veken et al. [32] showed that students attending an integrated medical curriculum reached a higher knowledge in basic sciences compared to their counterparts completing a conventional curriculum. According to focus group participants in the present study, knowledge retention is also promoted if contents known from school are repeated in the ISS courses. Such refreshment might be particularly important for students starting medical education a long time after attending science classes at school or college. ISS courses may thus counteract the loss of basic science knowledge during the course of medical education described by Custers [33]. Finally, students stressed the importance of applied exercises in the courses for knowledge retention. This is in agreement with results of a qualitative study in Canada, which identified integration of foundational and applied learning as a key issue for students’ knowledge retention [14]. Similarly, in the present study, students and lecturers outlined the integration of foundational and applied learning as a value of the ISS courses. Participants of both groups recommended such an integrative approach as a precondition for becoming a physician.

According to our qualitative results building close links between core curriculum classes (e. g., physiology) and ISS course contents is crucial for the success of the ISS concept. If medical students realize this connection an enhancement of motivation and engagement can emerge. On the other hand, this link still has to be improved in some courses, an aspect also confirmed by our survey: when asked whether ISS courses were helpful for preparation of core curriculum contents, students rated the courses differently, ranging from 3.5 to 5.5 on a 6-point Likert scale (with 6 as optimal possible value). In some cases, links to curricular topics were apparently not obvious for a large number of students. It will thus be necessary to intensify the collaboration between core curriculum teachers and ISS course teachers. Notably, both “building curricular links” and “interdisciplinary faculty collaboration” were also identified by Muller et al. [22] as major aspects of the establishment of an integrated medical curriculum. Moreover, the ability of lecturers to illustrate connections between ISS course topics and core curriculum topics was critical for students’ motivation, in line with a study stressing the importance of teachers’ facilitation skills in remedial programs [34].

Although rare, we found also critical remarks regarding the integrative concept of the ISS courses. Lecturers expressed the fear that the temporal distribution of ISS courses to several modules may threaten the systematic introduction of contents. Along this line, one might argue that principles of, e.g., chemistry, are understood best when taught en bloc. In contrast to this view, Norman [35], after having reviewed the relevant literature, concludes that spread learning is more helpful for knowledge transfer than en bloc learning. Interestingly, student focus group participants in the present study suggested that the partitioned way of presenting contents in the ISS courses indeed resulted in more intense discussions and improved knowledge retention.

What might be the specific value of teaching basic science contents at medical school instead of teaching them prior to medical school? Our qualitative findings give important hints concerning this question. First, lecturers and students unanimously stated that ISS courses contribute to achieving a basic understanding of science concepts. This maximizes students’ motivation and engagement immediately. In the long run, both groups expected this conceptual understanding to be critical for every physician. Second, an alignment of students’ proficiency level was identified as a major role of ISS courses, attaining the aim to take student heterogeneity into account. However, it was also claimed that the course program did not reach all students who might potentially have profited. Focus group participants suggested that the voluntary character of ISS courses might be one reason for that. At least for at-risk-students, Winston et al. showed that remediation programs work best if mandatory [34]. On the other hand, student focus group participants in the present study positively judged the voluntariness of ISS courses, promoting personal responsibility and self-organization. Furthermore, both, students and lecturers stated that voluntary participation leads to a selection of intrinsically motivated students, thereby positively influencing the work atmosphere in the courses. The voluntary character of ISS courses therefore appears to have both positive and negative effects.

In addition to “voluntary participation”, another theme emerging with regard to course setting was “teaching in small groups”. Students and lecturers agreed that the small group setup supported course success in various ways, including reduced inhibition, learner-centered flexibility and the formation of study groups.

Overall, quantitative data regarding student’s satisfaction with ISS courses support the generally positive view of ISS courses expressed in focus groups. However, there appear to be differences between individual course topics and classes. This might reflect the need to improve curricular links for certain courses, and to further optimize the selection of course topics. Based on Goldman’s and Schroth’s framework for levels of integration [36], the satisfaction at the program level seems to be high, however, some needs for improvement remain at the course and session level.

Our study is subject to some limitations. First, one might claim that student focus groups were not representative, as they included only students interested in the ISS course program, and thereby positively biased students. However, it should be noted that at least results concerning overall satisfaction with the courses were quite consistent across focus group participant questionnaire and general student survey. Second, statements on retention of knowledge should be quantified objectively. Third, the program was still under development, leading to some changes in the course program. Still we believe that limitations mentioned above were partly overcome by using multiple data sources [31]. In particular, because ISS courses employ a highly innovative and complex concept, the mixed methods approach is suitable [28].

## Conclusions

In summary, our findings indicate that temporal and content-based integration of supportive science courses into the medical curriculum is an innovative and valuable educational approach. It seems to facilitate students’ motivation and conceptual understanding, as well as their subjective experience of retention of knowledge. The present data suggest that teaching basic science principles should not only be an issue of premedical education. By contrast, their integration into the medical curriculum might prove to be highly beneficial for medical students, not only for those at risk regarding basic science knowledge.

### Availability of data and materials

As data sharing was not stipulated in the ethics application approved by the Dean of the University Medical Center Hamburg-Eppendorf, data for this paper will not be shared. We did not have consent from the participants to do so.

## Additional files

Additional file 1: End-of-module Questionnaire. (PDF 55 kb) Additional file 2: Adjusted means and confidence intervals (95 %) of the items preparation and satisfaction. (PDF 28 kb) Comparison of students’ ratings of ISS courses during the first study year (cohort 2013/14). The items “*The ISS courses were helpful for preparing teaching units” (* ***preparation*** *)* and *“All in all, I am satisfied with the ISS courses” (* ***satisfaction*** *)* were rated on a 1–6 Likert scale ranging from “strongly disagree” (1) to “strongly agree” (6). Adjusted means and 95 % confidence intervals of the items are given for individual classes and modules. (PDF 76 kb)

## Abbreviations

ISS: integrated supportive science courses
iMED: integrated medical curriculum at the University Medical Center Hamburg-Eppendorf (“Integrierter Modellstudiengang Medizin Hamburg”)
Intro: introductory week
ITS: introduction to science
A1, B1: modules of the iMED curriculum
