# Supplementary material for: Exploring the value and role of integrated supportive science courses in the reformed medical curriculum iMED: a mixed methods study
Source: BMC Med Educ. 2016 Apr 29;16:132. doi: 10.1186/s12909-016-0646-9 (PMC4851779; doi:10.1186/s12909-016-0646-9)
Supplement: Additional file 1: — End-of-module Questionnaire. (PDF 55 kb) [file 12909_2016_646_MOESM1_ESM.pdf]

Additional file 1

End-of-module Questionnaire: Integrated supportive science courses (ISS)

Item	Response Category
Did you attend at least one ISS course?	Yes<br>No
Which ISS courses did you attend?<br>*Multiple responses possible.*	Biology<br>Chemistry<br>Physics<br>Mathematics
The ISS courses were helpful for preparing teaching units.	Strongly disagree (1)<br>Disagree (2)<br>Somewhat disagree (3)<br>Somewhat agree (4)<br>Agree (5)<br>Strongly agree (6)
All in all, I am satisfied with the ISS courses.	Strongly disagree (1)<br>Disagree (2)<br>Somewhat disagree (3)<br>Somewhat agree (4)<br>Agree (5)<br>Strongly agree (6)
What is your gender?	Female<br>Male
What is your age?	0 - 99
