# Supplementary material for: Exploring the value and role of integrated supportive science courses in the reformed medical curriculum iMED: a mixed methods study
Source: BMC Med Educ. 2016 Apr 29;16:132. doi: 10.1186/s12909-016-0646-9 (PMC4851779; doi:10.1186/s12909-016-0646-9)
Supplement: Additional file 2: — Adjusted means and confidence intervals (95 %) of the items preparation and satisfaction. (PDF 28 kb) Comparison of students’ ratings of ISS courses during the first study year (cohort 2013/14). The items “The ISS courses were helpful for preparing teaching units” ( preparation ) and “All in all, I am satisfied with the ISS courses” ( satisfaction ) were rated on a 1–6 Likert scale ranging from “strongly disagree” (1) to “strongly agree” (6). Adjusted means and 95 % confidence intervals of the items are given for individual classes and modules. (PDF 76 kb) [file 12909_2016_646_MOESM2_ESM.pdf]

Additional file 2

Table 1. **Adjusted means and confidence intervals (95%) of the items "Preparation" and "Satisfaction"**

		Item "Preparation"			Item "Satisfaction"		
		n	M	95% CI	n	M	95% CI
**Biology**	Intro[a]	239	3.94	3.7-4.2	238	4.44	4.2-4.7
	C1[b]	79	**5.01**	4.7-5.4	78	**5.27**	4.9-5.6
**Chemistry**	Intro	252	4.21	3.9-4.5	254	4.78	4.5-5.1
	B1[b]	170	4.82	4.5-5.1	169	5.00	4.7-5.3
	C1	156	4.89	4.6-5.2	151	5.16	4.9-5.5
	D1[b]	79	**5.70**	5.3-6.1	80	**5.73**	5.4-6.1
**Physics**	Intro	262	3.88	3.6-4.2	265	3.81	3.5-4.1
	A1[b]	216	4.17	3.9-4.5	215	4.14	3.9-4.4
	ITS[c]	-[d]	-	-	149	4.23	3.9-4.5
	B1	154	**4.63**	4.3-4.9	154	**4.67**	4.4-5.0
**Mathematics**	Intro	210	3.70	3.4-4.0	221	4.13	3.9-4.4
	ITS[a]	-[d]	-	-	273	**5.46**	5.2-5.7

[a]Intro: introductory week; [b]A1, B1, C1, D1: modules of the iMED curriculum (see figure 1); [c]ITS: introduction to science; [d]the item preparation was not part of the evaluation survey.

Figure Legend

Comparison of students' ratings of ISS courses during the first study year (cohort 2013/14). The items "*The ISS courses were helpful for preparing teaching units*" (***preparation***) and *"All in all, I am satisfied with the ISS courses"* (***satisfaction***) were rated on a 1-6 scale ranging from "strongly disagree" (1) to "strongly agree" (6). Adjusted means and 95% confidence intervals of the items are given for individual classes and modules.
